# Eco-evolutionary perspectives on emergence, dispersion and dissolution of historical Dutch commons

**DOI:** 10.1371/journal.pone.0236471

**Published:** 2020-07-30

**Authors:** Anders Forsman, Tine De Moor, René van Weeren, Giangiacomo Bravo, Amineh Ghorbani, Molood Ale Ebrahim Dehkordi, Mike Farjam

**Affiliations:** 1 Department of Biology and Environmental Science, Linnaeus University, Kalmar, Sweden; 2 Department of History and Art History, Utrecht University, Utrecht, The Netherlands; 3 Department of Social Studies and Centre for Data Intensive Sciences and Applications, Linnaeus University, Växjö, Sweden; 4 Faculty of Technology, Policy and Management, Delft University of Technology, Delft, The Netherlands; Indiana State University, UNITED STATES

## Abstract

Historical commons represent self-governed governance regimes that regulate the use and management of natural and man-made shared resources. Despite growing scientific interests, analyses of commons evolution and temporal dynamics are rare and drivers of change (birth, adaptation, dissolution) remain obscure. We apply an interdisciplinary approach and address these issues from an eco-evolutionary perspective. Analyses of > 400 Dutch commons over more than a millennium (between the 9^th^ and the 20^th^ century) uncovered that most commons originated between 1200 and 1700, and that there was a particularly high rate of evolution during 1300–1550, a pattern intermediate to gradualism and punctuated equilibrium in biological evolution. Dissolutions of commons were rare prior to 1800 and peaked around 1850, comparable to a mass extinction in biology. Temporal trends in number, spatial distribution, density, and dispersion of historical commons were distinctive and resembled developments seen at the levels of species and individuals in the growth of biological communities and populations, in that they showed signs of saturation determined by the abundance and distribution of resources. The spatiotemporal dynamics of commons also pointed to important roles of social, economic and political factors, such as new reclamations of resources and pressure on resources due to population growth. Despite internal and external pressures, the self-governing commons studied here were very successful, in the sense that they persisted for on average >350 years. There was a weak positive relationship between the use of multiple resources and the lifespan of commons, resembling associations between diversity and persistence seen in biological systems. It is argued that eco-evolutionary perspectives can further the understanding of the long-term dynamics of commons as institutions for collective action, vitalize future research, improve management of shared goods, and advise about sustainable utilization of finite resources.

## Introduction

Throughout history, self-governed commons have been organized to manage the use of natural and man-made common-pool resources by a group of users [1, 2]. In this context, institutions represent the common property systems of agreements, rules and enforcement mechanisms aimed at coordinating and regulating both the commoners’ sustainable consumption of limited shared resources and their provision of labour and investments for the commons’ maintenance and long-term well-being. In our study, commons are considered as institutions for collective action [1], and as such the term commons does not simply refer to the resources held in common but also to the organisation that is run by its members. When users collectively decide to manage and use a resource jointly, a social dilemma emerges that can be solved by designed rules on access and appropriation. These rules are written down and the common becomes considered as an organisational body, an “institution”, that is responsible for the execution of the rules [3, 4]. The set-up of such institutions for collective action has been directed towards collectively beneficial outcomes whilst overcoming conflicts of interest and potential selfish short-term temptations associated with social dilemmas, also referred to as the Tragedy of the Commons [5–9]. In the Tragedy of the Commons it has been suggested that management of resources cannot be sustainable in a collective action setting because of incentives to behave selfishly linked to the structure of the situation. Amongst others the work by Nobel-winner Elinor Ostrom has delivered empirical findings pointing to the capacity of commoners to develop robust institutional frameworks that allow for long-term sustainable resource governance [1]. However, despite a growing scientific interest in the long-term development of commons-institutions [10–14], and the general acceptance that commons do evolve over time [11], important aspects of their evolutionary dynamics and the underlying drivers remain largely unexplored [15].

Transdisciplinary thinking has spurred the growth and advancement of biological thought, as exemplified by the assimilation of socioeconomic reasoning regarding population growth [16] by Charles Darwin when developing the theory of common descent and evolution by natural selection [17]; by the unification of statistics, genetics, and selection theory under the modern synthesis of evolution in the 1930s and 1940s [18]; by the incorporation of game theory in the analyses of behaviours in animals and humans [19]; and by recent developments in bioinformatics analysis owing to advancements in computational techniques for big data handling [20]. In a similar vein, theories and approaches developed in ecological and evolutionary research have potential to further understanding and vitalize research in social sciences and humanities in general [21], and to inform about the evolutionary dynamics and developments of business organisations and policy agendas [e.g., 22, 23–26], and of historical commons in particular [e.g., 15, 27, 28, 29]. With regard to biological analogies, historical commons may be thought of as representing individuals, populations or species, depending on the context, and in many cases the driving factors and expectations from theory are sometimes similar for these different hierarchical levels of organization.

Our approach in the present study is inspired by and parallels the work done on organizations during the 1980s and 1990s within the framework of organizational ecology [22–24]. However, while organizational ecology focused on the social environment as the main selective force leading to the evolution of organizations, we adopt here an approach where social, institutional and ecological factors jointly interact to explain the observed outcomes. This is consistent with the broader perspective of social-ecological systems, seen as complex dynamic systems where biophysical and social factors interact to form a consistent a resilient unit [3, 4].

In this study, we analyse patterns of the origination, long-term developmental modifications and dissolutions of self-governed historical Dutch commons that were established for the sustainable utilization of shared and collectively managed resources. Within ecology and evolution, speciation, evolutionary modifications and adaptations, and ultimately extinctions have been analysed to inform how environmental factors and species characteristics drive the spatial structure and temporal dynamics of biological diversity [30–33]. Similarly, the development of the institutional design of commons (rules, regulations and decision-making processes) is likely reflective of both internal and external factors, which in some periods remain rather stable, and in other periods change rapidly.

The commons included in our study are part of an area that is relatively homogenous in landscape, resource distribution, population density and urbanisation rates, and that has until the end of the 18^th^ century experienced relatively little change in population growth or resource exploitation. The type of Dutch commons involved, the so-called *marken*, was a typical form of an institution for collective action [1] for the northern and eastern parts of the Low Countries during the medieval and early modern period, until the middle of the 19^th^ century, when all over Europe the existence of commons was challenged by developing nation states. Examples of potentially important intrinsic or internal factors include the nature and number of the shared resources, and the number (and personality) of the commoners. The soil in these areas was difficult to cultivate for growing crops because of the presence of extensive peat bogs and marshlands, with a relatively low population density (even despite the population increase in this area between 800 and the 18^th^ century) as a consequence (see Results for details). Peat was one of the main resources to be found on the common land, but as it was a depletable resource, its’ harvesting for use as fuel was often restricted. The potential depletion and shortage of this resource was a course of concern for every commoner as few other alternative forms of heating were available on a large scale in that period. Attempts to cultivate the area on a large scale did not take of in this region until the second half of the 19^th^ century. Although the land was formally owned by civic or ecclesiastical rulers, the day-to-day management and governance of resources often was in the hands of its users, whereby their entitlement to use the resources was usually linked to the ownership of a specific farm or estate. Due to this entitlement system, the number of users within the common remained relatively stable over time. Population growth in the 19^th^ century, in particular linked to reclamation projects, consisted mainly of impoverished families, who were attracted from other regions in the area to work as journeymen and barely survived as cottagers. Further development of infrastructural works, strongly supported by the Dutch King William I, also facilitated the transport options to and from the formerly undisclosed areas of the Low Countries [34].

External factors that have influenced the demography and resource use in the area include in particular political (political turmoil, warfare) and environmental conditions and events, such as disease, natural disasters and climate change. In the (S1 Table), we provide an overview of the various threats that we could trace for this particular area that might have influenced the commons under study for a period starting a few centuries before we see the first foundation acts of commons being registered until the middle of the 19^th^ century when commons in this area disappeared by and large. Besides the complexity of cultivation, the risks of war and other external events, and the risk of overuse of resources, natural causes also could be a threat to the scarce resources within the commons. The largest threats came from river floods (often caused by storm surges over the Zuiderzee), making both pasture land and peat bogs unusable and cattle diseases: two waves of cattle plague in the 18^th^ century literally decimated the number of livestock, killing respectively about 90 and 70 percent of all cattle in the Low Countries. Cattle diseases may have had a profound effect on the usefulness of the commons for their members, in particular if pasture land was one of the available resources used.

Elsewhere, it has been demonstrated that the reduced usefulness of the common to its members may affect the willingness of commoners to preserve their common as a collective resource [6]. Besides cattle diseases, general impoverishment of the population may also reduce the cattle possession amongst farmers and thus lessen the interest of commoners in the pasture land. This in turn may have resulted in them being less willing to fight for the preservation of their common rights [35] and a higher vulnerability to factors—such as government plans to dissolve the commons—that eventually can lead to their mass extinction.

Applying explanations and analytical approaches used in studies of evolutionary dynamics of biological systems to studies of the evolution of institutions for collective action such as commons is justified because, similar to biological individuals, populations, species, and organism communities, commons function on the basis of internal self-regulation and respond to external pressures via adaptation (of the regulation) or disappearance. Contrary to organizations that are private (and thus run by a limited number of individuals having an impact on the course of the organization) or public (whereby the state coordinates the changes and evolution of the organization), commons depend highly on self-organization and cooperation by most members of the group in order to ensure the organization can survive. Despite the potential benefits, attempts to quantitatively study patterns and identify drivers of institutional dynamics such as those of commons remain scarce [10, 14, 27, 36]. One main reason for the rarity of such studies is the scarcity of data that would allow for identifying long-term patterns in institutional dynamics. Although the archival material left to support such studies is often sufficient, the transcription and processing of such data is extremely labour intensive. However, for particular geographical areas, sufficient data is now available to study such dynamics, allowing us to explore potential cross-fertilization between evolutionary biology and studies on institutional dynamics. For the first time, it can be empirically shown that the cultural evolution exhibited by Dutch institutions follows patterns similar to those detected in biological populations and species.

Two long-standing debates in evolution concern whether speciation events and extinctions have occurred at constant rates (gradualism) or whether there have been shorter bursts with elevated speciation alternating with periods of stability (punctuated equilibrium) [37], and on the importance of background versus mass extinctions in relation to large scale geological events and evolutionary key innovations that drastically changed the conditions for life [18, 38–40]. In political sciences, punctuated equilibrium has been proposed as a pattern explaining changes in outputs of governmental processes and policy agendas [25, 26, 41]. To our knowledge, it has not been evaluated whether origination and dissolutions of commons throughout history conform to any of these patterns in biological evolution. We consider here commons as organizational units within socio-ecological systems [3, 4, 42], and propose to draw parallels on a more abstract level between evolutionary developments of ecosystems and those of commons [43].

We look explicitly at the origins (“birth”) and dissolutions (“death”) of commons as individual organisational units that were part of a wider “population” of commons in the area. In this paper, we do not explore the potential similarities between species in evolutionary biology and the internal changes of commons as individual institutions. Extinction, in the context of socio-ecological systems, refers here to a transformation of the common in a private-or publicly managed system of previously collectively held resources. In European history, the “extinction” of commons has been described as the dissolution of the organisational units both in a legal and physical sense: the legal rights to manage and use the collective resources were taken from the commoners, either through privatisation (sale of the resources) or through usurpation by the government (for which, sometimes, commoners were compensated). The latter procedure, which eventually did lead to massive sale of the formerly common land, took place all over Western Europe in the period 1750–1900 [44–46].

In biology, ecological and behavioural factors together with resource availability influence spatiotemporal dynamics of the number, distribution, density, and dispersion of species, individuals, and territories. For example, in community ecology, changes in number of species that occur in an area reflect a balance between rates of colonization and extinction; and the equilibrium species richness depends on species characteristics, resource availability, and environmental heterogeneity [47]. In population biology, temporal changes in the number of individuals are determined by the per capita birth-death rates, and limited by the carrying capacity of the habitat via environmental resources (i.e., logistic population growth [48]). In behavioural ecology, the pattern of spatiotemporal settlement and dispersion of breeding territories in birds and of nests in social insects such as ants, bees and wasps can be influenced by the spatial distribution of resources, individual qualities and by the outcome of competitive interactions [49–52]. Clumped or aggregated patterns of dispersion (of species, individuals or territories) may result if resources are not uniformly distributed but disproportionally concentrated in small areas within a larger habitat or region, uniform or evenly dispersed patterns may be reflective of interactions between individuals such as competition and territoriality, and random dispersion patterns might indicate that interactions among individuals (or species) are unimportant, or that the distribution of resources is random.

Here, we first examine whether the large scale patterns of births (emergence/origination) and deaths (dissolutions) of historical commons conform to the patterns of speciation and extinction in biological evolution. To that end, we analyse temporal distribution of origination and dissolutions of > 400 Dutch commons spanning in total more than a millennium, from 800 to ca 1900, in a fairly concentrated area in the east of the Netherlands, using data collected within the framework of the “Common Rule(s)-Project” (CRP) [12] (see Methods). Next, we perform more in-depth analyses of the spatiotemporal distribution dynamics of commons. Specifically, we examine how the number, spatial distribution, density and dispersion of commons changes over time. Finally, we evaluate whether differences in the number of resource types used is associated with variation in the lifespan of commons.

## Results and discussion

### Births of commons

The CRP-database comprises over 800 commons that emerged in the same period and region, but information on both start and end date is not available for all [12]. Year of births is determined based on information that can be retrieved from the written sources that were still available in the historical archives of the region; arrangements may have existed also elsewhere already, but may not have been written down, or written agreements may have been lost over time. Births (*n* = 342) and deaths (*n* = 415) were therefore analysed for a subset of Dutch commons with both the year of origin and of dissolution available for cases in between the 9^th^ and 20^th^ century (see Methods). Given that this is historical research, it is possible that some cases that were shorter-lived did not leave traces in the archival documentation available for the area, and thus were not included in our analyses. Although this may have some effect on the average longevity of the commons, we do not expect that a major incidence of dissolution/extinction of commons would have remained unrecorded in the archives, nor do we observe a specific gap in the presence of commons that may point towards a loss of archival information for a specific area. Given that we are focussing here for the larger part on a period with only limited change in natural resource use and population growth, we do not expect that the number of resources available per common -which is a parameter used in some of our analyses below- would have changed substantially throughout this period. Because of the limited fertility of this area (see earlier in this article), the potential for adding other resources through for example intensive cultivation of specific crops would have been limited.

**Fig 1:**
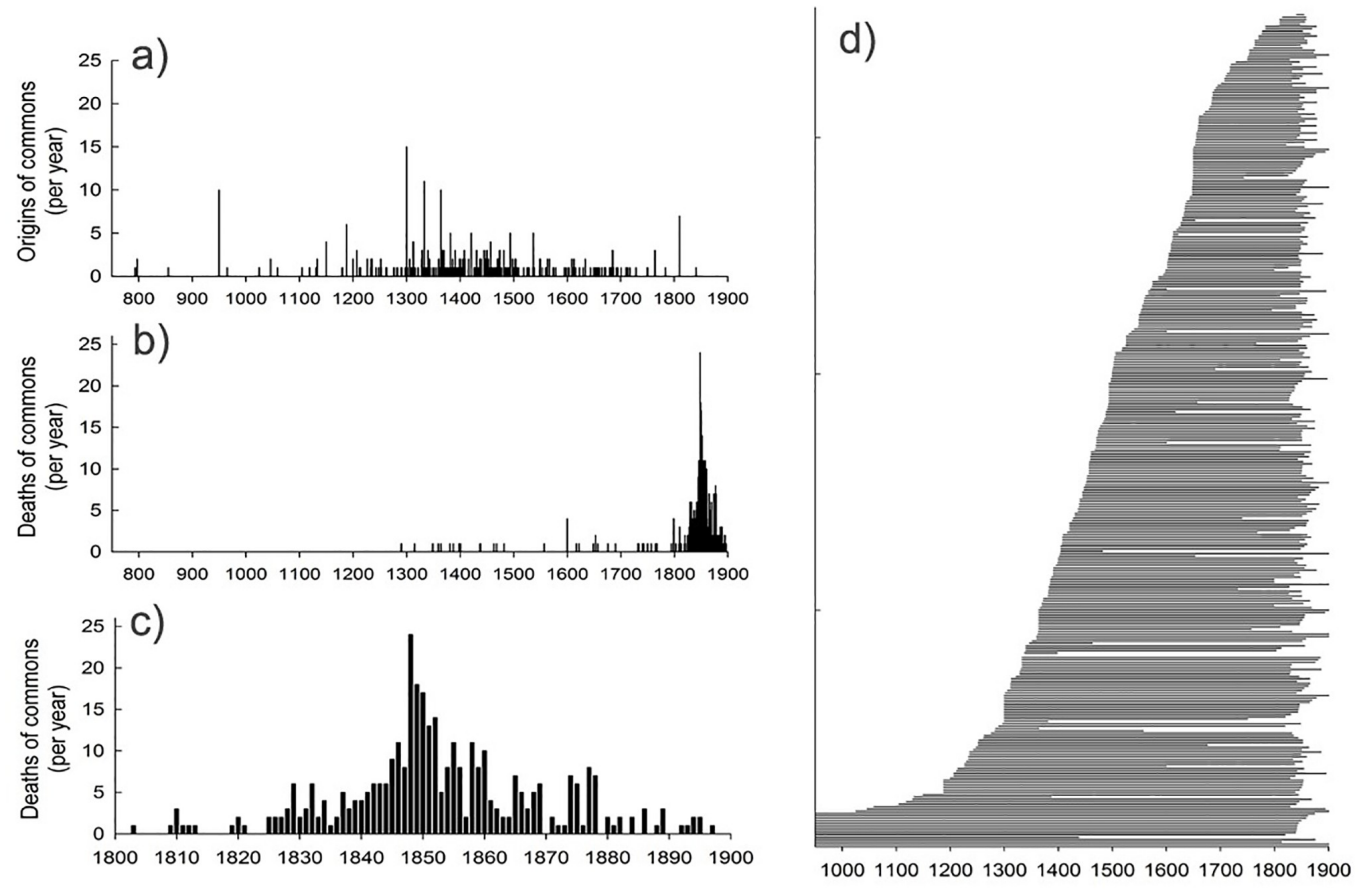
Temporal evolutionary dynamics of historical Dutch commons. Births or origins (a, *n* = 342 commons), deaths or dissolutions (b and c, *n* = 415 commons), and lifespans (d, *n* = 351 commons) based on known or estimated year of birth and dissolution for Dutch commons.

The distribution of origins of commons points to a pattern that is an intermediate to that of gradualism and punctuated equilibrium in biology [18, 37]. Origins of commons were relatively frequent between ca 1200 and 1700, with a distinctive peak at the end of the 10^th^ century, a more prolonged period with a high rate of evolution between ca 1300–1550, and yet another peak at the beginning of the 19^th^ century (Fig 1a). New commons were initiated at a somewhat slower rate after ca 1500 (Fig 1a). As discussed in greater detail below, it is plausible that this reduced speed was due to saturation. Most villages would have had a common by then, but once commons had been set up there would have been little need and limited opportunity to establish additional ones in the same area. Similar patterns of sequential habitat selection and territory establishment, driven by the interactive roles of environmental properties and competition, have been reported in birds, [53, 54].

### Deaths of commons

Although they may have left comparatively few traces in historical archives, it is very likely that dissolutions of Dutch commons were fairly rare prior to 1800. The majority was dissolved during the 19^th^ century with a clear peak around 1850 (Fig 1b–1d), as the result of the legislation that was putting pressure on commoners to privatize their commons [44, 46, 55] (see S2 Table). This is in line with a European-wide trend of dissolving most commons, in particular in North Western Europe [44, 45]. The resources held in common were then, depending on local arrangements, partitioned among previous right holders (commoners), or sold in their entirety or parts to a private party, or transferred to the local government who then treated it as public property. Collective self-governance by the users of the land was however, from then on, no longer the case, even in those cases where commoners managed to obtain part of their common. Whenever such partitioning, sale or transfer took place, we can thus safely say that the common as a specific self-governing organisational unit had been dissolved.

### Lifespans of commons

Importantly, our dataset on commons in eastern Netherlands shows that—contrary to expectations following out of the literature based on the Tragedy of the Commons [7]–commons in the past overall were long-lived (mean ± s.d. = 371 ± 200 years, range 9–1110 years, Fig 1d). Commoners were capable of developing a solid institutional structure that allowed them to avoid freeriding having a detrimental impact on the resource use. Moreover, our data also show the processes of change commons went through in the long run. The commons in our dataset went through a fairly abrupt dissolution process, which shows resemblance to the great mass extinctions in biology [18, 38]. As in biology, the dissolution of historical commons was also induced by external factors [56]. In the period of mass dissolution, increasingly stringent top down national legislation was introduced (S2 Table) aiming at abolishing all forms of collective resource management in order to stimulate the reclamation of uncultivated land [6, 57]. This was driven by the belief that privatization would lead to reclamation and subsequently enable upgrading the land into higher economic value, better profit making opportunities, and more efficient management and utilization [58].

All commons in the Netherlands were subject to the legislation of 1810 by Royal Decree that sought to divide and sell the uncultivated lands. In many cases, such as in the case of the common Raalterwoold,—one of the more thoroughly studied commons in our database [59], implementation of this decree caused a debate among the commoners, some of whom wanted to implement the division and sale of land to their benefit, others however did not see how such a division would be to the benefit of (the majority of) the commoners. Raalterwoold was a common that did not, contrary to most other marken in the region, coincide with the area belonging to the jurisdiction of a village, but covered an area that comprised several small villages. In early medieval times, the area of Raalterwoold was one of the largest uncultivated areas in the eastern part of the Netherlands [60]. Research on other areas of the Netherlands Slicher van Bath 1944 [61, 62] (also mentioned in [60]) indicates that from the thirteenth century on the management of large areas of uncultivated land was taken up by small groups of farmers. Possibly, mutual agreements between landlords and local farmers had existed even before the first (preserved) written regulation. When the government ordered the division of the marken in the 19^th^ century, the Raalterwoold commoners pointed out that most of the uncultivated lands had already been developed or used for the construction of roads etc.; division of the remaining lands that had not already been used for road construction among the owners of shares would result in less than half an hectare (*bunder*) per share, an amount they considered as being ‘never a reasonable compensation for the [loss of] existing rights of mowing and grazing’ [63]. As the government levied high taxes on uncultivated land, the tax burden on the common subsequently increased. Whereas in earlier times it was customary for tax collectors (who were held personally responsible for sufficient tax income derived from the use of the common) to supply money from their own pocket to solve an eventual deficit at the end of the year and deal with any debtors personally afterwards, the increased tax burdens made this hands-on solution no longer possible, making the sale of land the only realistic option. As the remaining land had already been sold to solve the debts of the common, it was ordered in 1841 to sell most of the remaining uncultivated land. Many of the commons experienced a similar process, causing a ‘mass extinction’ of Dutch commons throughout the first half of the nineteenth century [64].

### Changes in number, spatial distribution, density and dispersion of commons

**Fig 2:**
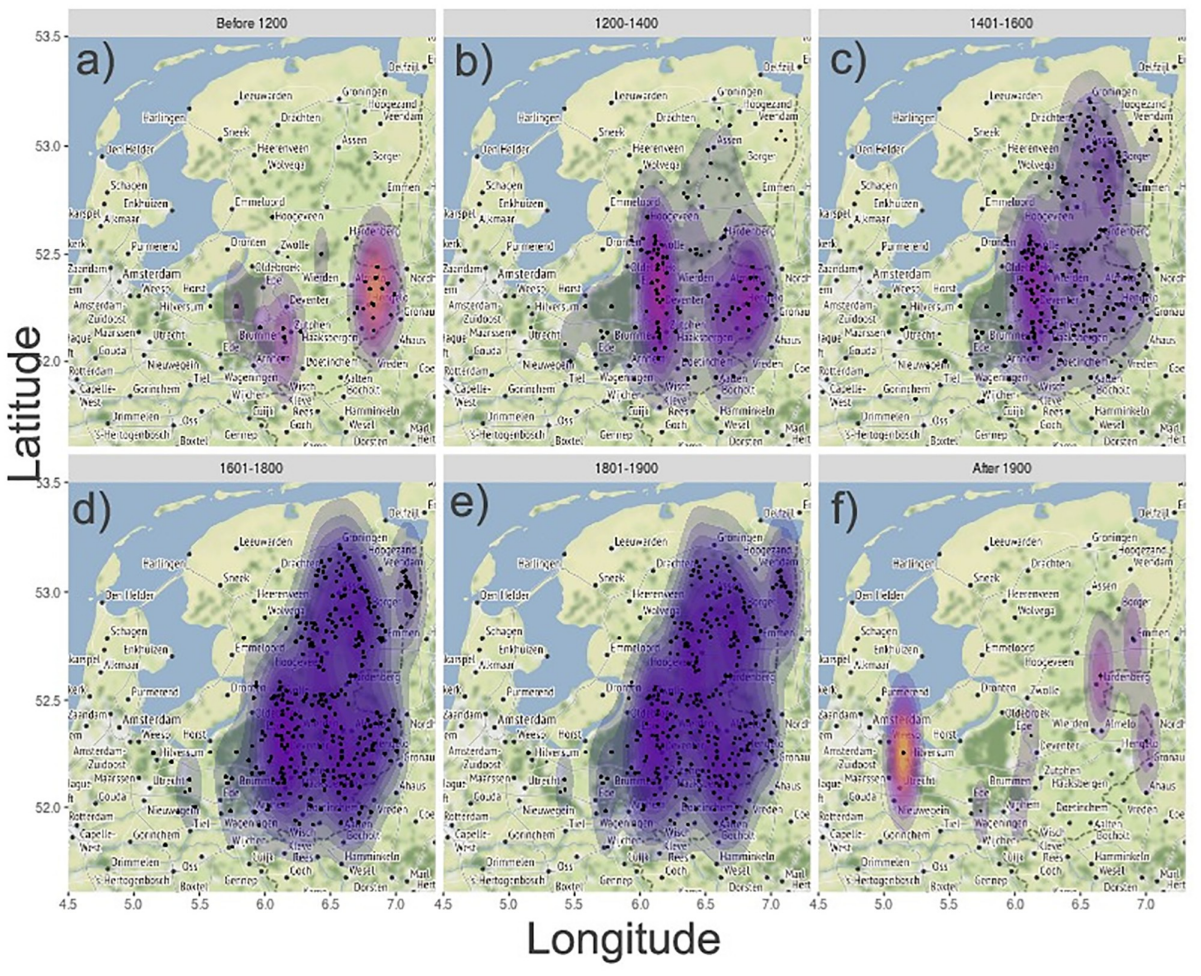
Change in spatial distribution of historical Dutch commons from the 13^th^ to the 20^th^ century. The figure shows the distribution, dispersion and density of commons (**a)** before 1200, **b)** 1200–1400, **c)** 1401–1600, **d)** 1601–1800, **e)** 1801–1900, and **f)** 1901-). Each black dot represents one common. Shaded areas represent 2D kernel estimations of commons density. Note: commons are plotted on a map representing contemporary, not historical terrain. Map tiles by Stamen Design, under CC BY 3.0 Data by OpenStreetMap, under ODbL.

**Fig 3:**
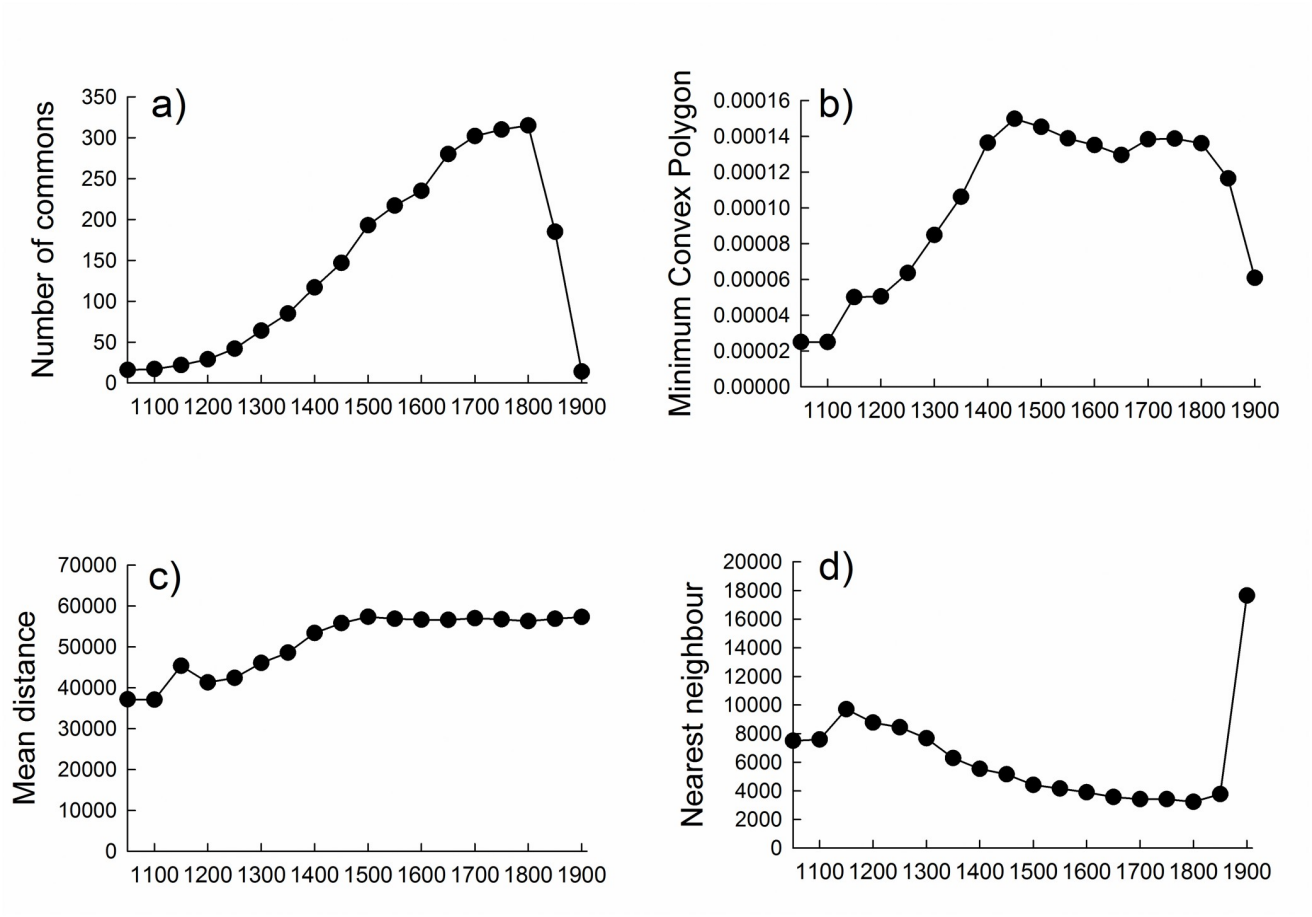
Changes in number, spatial distribution, density and dispersion of historical Dutch commons over time. Temporal trends, shown for 50yr intervals, in **a)** number of commons, **b)** total area (Minimum Convex Polygon) occupied by commons, **c)** mean distance between commons, and **d)** mean nearest neighbour distance between commons. Figure is based on information for commons with known or estimated year of birth, year of dissolution and geographic location.

There were distinctive temporal trends in the number, spatial distribution, density, and dispersion of historical Dutch commons (Figs 2 and 3, S1 Movie).

**Fig 4:**
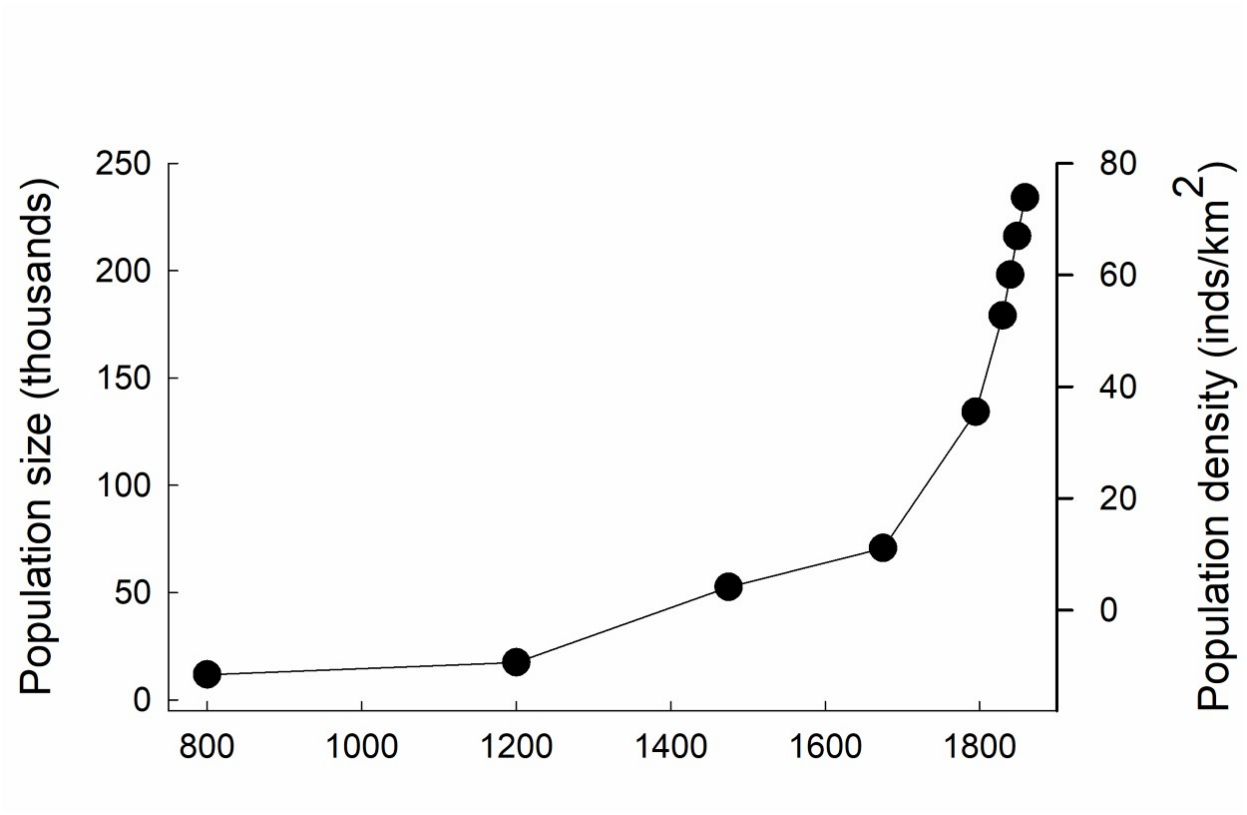
Population growth and density in the Overijsselse region during ca 800–1859. Data on population size and density are from table on page 95 in Slicher van Bath (62). Data for the period between 1795 and 1859 are based on Dutch censuses [67].

The number of commons simultaneously alive within the studied area increased in a sigmoidal manner from less than 50 to more than 300 during the period 1100 to 1800, and then rapidly declined during the 19^th^ century (Fig 3a). This development closely resembles patterns driven by competition for limited resources seen at different organizational levels in biology, such as the establishment and gradual accumulation of new species in communities on islands towards an equilibrium species richness [47], or the growing number of individuals in natural populations towards carrying capacity of the environment [48]. The earliest commons appear in the areas where the oldest permanent settlements were established, along the smaller rivers in the provinces of Overijssel and Gelderland (Fig 2). The human population in this region grew substantially during the medieval period, from < 4 individuals/km^2^ around year 800 to ca 70 individuals/km^2^ in the middle of the 18^th^ century (Fig 4), as elsewhere in Europe [62]. With the increasing pressure on resources due to population growth, there was a growing need to lay down rules on the use and management of resources in written agreements. It is generally acknowledged by historians, that population growth put great pressure on the fragile mixed agricultural system, whereby pasture land played a vital role but was also the most likely to be reclaimed and turned into arable, to feed the growing population [65]. Keeping a balance between arable land and pasture land—needed for the manure provided by the cattle—was however of vital importance for the productivity of the crops. Increasing peat reclamation may have led to population growth via immigration, at least from the 16^th^ century onwards, but rapid population growth did not occur until the establishment of large peat reclamation projects in the area in the 19^th^ century [66]. Peat was for the major part exported outside of the region to serve as fuel. Instead, the pressure on pasture land may have been higher than on peat in the communities where new commons were set up. In essence, there was probably a trade-off between conflicting interests and demands, whereby pasture land may have served as the resource to be protected, rather than protecting the peat land.

The total area occupied by commons, as estimated using the minimum convex polygons [40, 68], expanded rapidly from the 12^th^ to the 15^th^ century and then remained relatively stable until around 1800, after which it declined again (Figs 2 & 3b). The number of commons continued to increase also after the geographical expansion phase had come to an end. From 1400 onwards, new commons no longer emerged in isolation far away from other commons—they were instead established in closer proximity to and between already existing commons, resulting in an increased density of commons. In support of this interpretation, the mean distance between commons increased up until about 1450 and remained stable thereafter (Fig 3c), whereas the mean nearest neighbour distance declined at a progressively slower rate throughout most of the study period (Fig 3c). These spatiotemporal trends in the establishment and dispersion of commons (Figs 2 & 3), which are at least superficially similar to the sequential occupation and distribution of breeding territories in heterogeneous environments seen in some species of birds [49, 50], are indicative of saturation.

From the historical literature there are so far no indications that the decisions to set up a common as a way to govern resources was a coordinated action by local or supra-local authorities, or that this solution was actively copied among commoners. More likely, villagers came to the conclusion that an alternative to the open access-situation, which would have been dominant before, was needed to secure resources from being overused due to a lack of clear property rules, which would also include regulation of access and use [1]. The spread of commons after 1400 can be easily be explained by the settlement of new villages in between others that also wished to set physical and legal boundaries to their resources to prevent people from neighbouring villages from using them. An alternative dispersal method would have been that as villages grew so did the area of the associated collectively owned resources. However, we are not aware of any example of a common that became larger over time by additional acquisitions of land, though some commons merged into larger ones (e.g. the Marke Verwolde, Laren, and Oolde) that orginated from the merge of three smaller commons (*buurtschappen*) of these separate villages [69]. Although the massive dissolution of complete commons did not take place until the 19^th^ century, commoners often had a hard time in defending their resources from other parties interested in claiming them. For example, the sale of plots of land near ‘s-Graveland, belonging to the common Het Gooi, to Amsterdam merchants by the provincial authorities (Staten) of Holland in 1625 without proper compensation to the commoners of Het Gooi for the loss of usable common land, lead to fights and compensation claims [70].

The distinct drop in the number of surviving commons that occurred in the 19^th^ century (Fig 3a) was caused by an increase in the rate of death or dissolution of commons at this time (Fig 1b and 1c) and lead to a marked decline in the area occupied by commons after 1800 (Fig 3b). This temporal development shares a strong resemblance to the population declines and range contractions seen prior to the extinction of biological species and populations [31, 40]. The long-term pattern, with regards to the rise, dynamics and fall of historical Dutch commons, documented in this study is best understood as the result of a complex interplay of ecological, social, economic, and political factors and processes (summarised in S1 and S2 Tables). Resource protection through the setting of legal boundaries—by defining who could use the resources and to what extent—was vital to limit overexploitation of the resource under open access conditions which could eventually lead to a self-inflicted destruction of the common.

### On the relationship between multiple resources use and lifespan of commons

**Fig 5:**
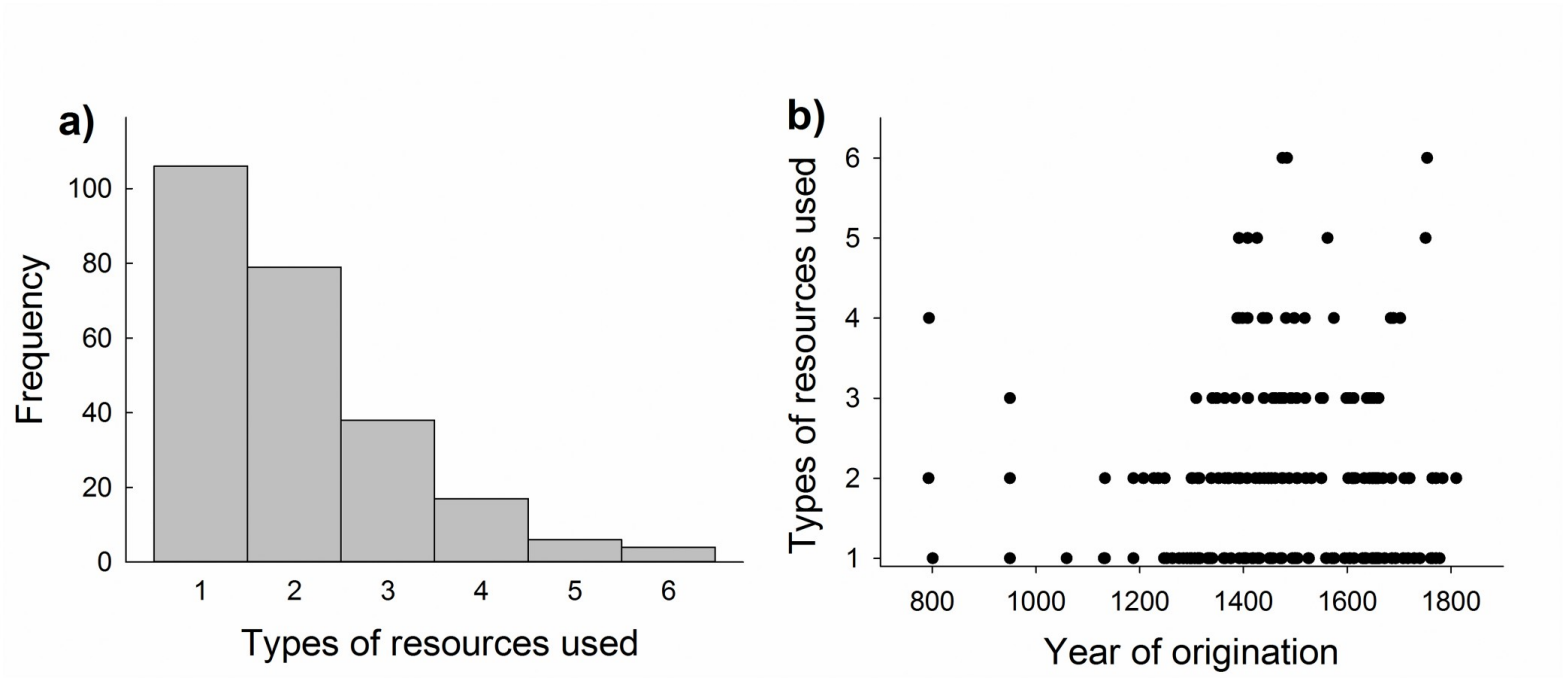
Number of resource types used by historical Dutch commons. a) Absolute frequency distribution. b) Number of resource types used in relation to year of origin of the commons.

**Fig 6:**
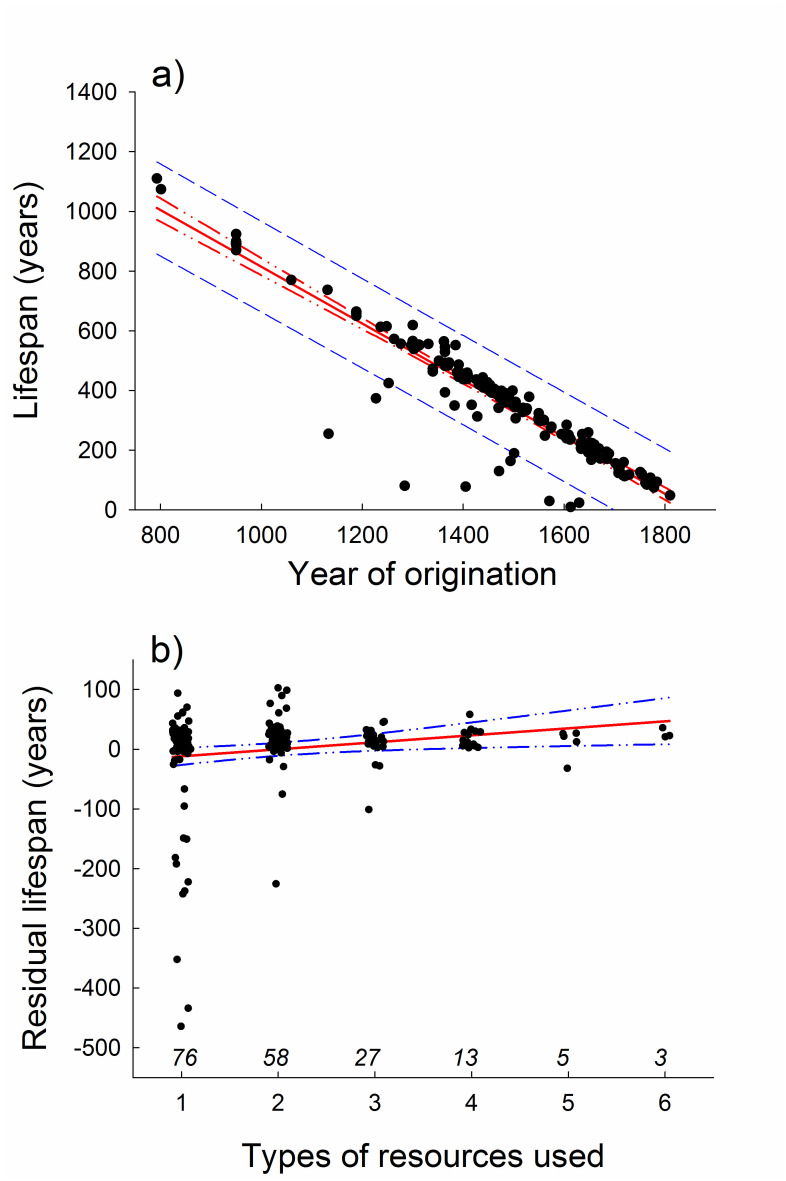
Lifespan of Dutch commons as a function of year of origination and number of different resource types used. a) Total lifespan of commons in relation to year of origination. b) Relationship between residual lifespan (calculated as residuals from the least-squares linear regression fitted to the data shown in the top panel) and the number of different types of resources used by commons. Values in italics above the x-axis represent sample sizes. Data on number of resource types have been jittered along the x-axis, but statistical analyses were based on the original data.

Our analyses suggest that the use of multiple resources improved survival rates of historical Dutch commons. Most (ca 75%, 134 of 182) of the commons for which information was available in the historical records used only one or two different resource types, and none used more than six resources (Fig 5a). The number of resource types that could be found on the commons was restricted to what was naturally available. Examples of shared resource types that were used and regulated by the commons include forest, heath, meadows, pasture, crops, grass, wood, fish, peat, clay and drifting sand. In general, commons that originated later tended to rely on a larger number of resource types that were collectively managed via rules and regulations compared with commons that were established earlier (Fig 5b). The lifespan of commons declined with year of origination (Fig 6a) and increased in a linear manner with increasing number of resource types used (as evidenced by the results from multiple regression analysis, Table 1, Fig 6b). The estimate for the quadratic effect of resource use was negative but non-significant (*p* = 0.085, Table 1), suggesting that the benefit of multiple resource use in terms of survival might follow the law of diminishing returns. However, results of the randomization test support the conclusion that the quadratic effect was not significant (*p* = 0.15 (153/2000)). On the other hand, the evidence for a positive linear association of multiple resource use with residual lifespan (i.e., after controlling for the effect of year of origination) of commons was robust (Fig 6b), being statistically significant even when evaluated using non-parametric Spearman rank correlation analysis (*r*_s_ = 0.16, *n* = 182, *p* = 0.035).

**Table 1:** Lifespan of commons in relation to year of origination and resource use.

Variable	*df*	Parameter Estimate	Standard error	*t*	*P*
Intercept	1	1719.7	45.62	37.70	< .0001
Year of origination	1	-0.96	0.027	-34.63	< .0001
Resource types	1	41.7	17.71	2.35	0.0198
Resource types^2^	1	-5.4	3.10	-1.73	0.0851

Associations of survival (lifespan) of historical Dutch commons with year of origin and the number of resource types used as revealed by multiple regression analysis. Resource types and resource types^2^ (squared) represent the linear and quadratic effects, respectively.

The above finding adds further support for variation and generalization as key to success, and shares resemblance with the conclusion from studies in biology that diversity and generalization can offer advantages over specialization via a variance reducing portfolio effect that improves the ability to cope with changing and novel conditions, increases resilience, and allows for long-term persistence [71–73]. In general, the effect of diversity on persistence is seen in biological populations and species, historical commons, and in contemporary households [74], service providing organizations [75], and commercial companies, manufacturers and retailers [76–79]. It thus represents an additional analogy between the dynamics of biological and social systems.

## Conclusions

Our findings provide new knowledge of the evolution and dynamics of commons institutions management of shared resources. The temporal pattern of origins of commons is intermediate to gradualism and punctuated equilibrium seen in the evolution of new species, whereas the pattern of dissolutions of commons resembled a mass extinction in biology. The changes over time in the number, distribution, dispersion and density of commons were distinctive and resembled developments seen at the levels of species and individuals in the growth of biological communities and populations, in that they showed signs of saturation determined by the abundance and distribution of resources. The spatiotemporal dynamics of commons also pointed to important roles of social, economic and political factors, such as increased demographic pressure, an increasing number of settlements in the area, and economic development through for example commercialized peat reclamation. Despite internal and external pressures, the self-governing commons studied here were very successful in terms of survival, as they persisted for a period of on average more than 350 years [see also 12, 59, 80].

In this study, we also looked at the interaction between the end of their lifetime and the external conditions that contributed to their “mass-extinction”. The dissolution of the commons in the mid-19 century points towards the idea of *institutional friction* [25], with political factors external to the commons themselves progressively undermining their legitimacy and economic resilience and finally leading to their mass extinction. One of the explanations for the rapid extinction of the commons is the overall lack of cooperation between commons to organise their defence against top-down decisions to dissolve them. Their lack of coordinated defence against these governmental actions made them highly vulnerable [81].

Our study exemplifies how eco-evolutionary perspectives and interdisciplinary approaches can further understanding of the long-term development of commons and other types of institutions for collective action on a local level. It seems likely that continued exchange of ideas, methodological approaches, and insights have potential to vitalize future research and promote common knowledge building. Our findings have policy relevance for current challenges associated with a growing number of institutions for collective action (ICA) that are being set-up across Europe, and for the remaining ICAs across the world that are constantly threatened by privatization of resources. For the currently developing new wave of such institutions [82], the capacity to survive extremely long demonstrated by historical commons can offer guidance on institutional design and highlights the factors that may endanger their survival [6]. Although it is clear that commons can survive for several generations, we have shown that they were highly vulnerable to external pressures, such as a specific legislative culture, and that the use of multiple resources offered protection in terms of increased survival. Given the popularity of commons as an alternative governance regime in public and private contemporary resource governance, the realization that ‘commons are easy to destroy, but hard to grow’ requires careful attention by governments across Europe today.

The joined efforts by citizens who try to economise the use of resources by joining forces may be an alternative solution that offers better opportunities to self-limit resource use than market or government organised resource use. Our results suggest that regardless of the efforts it takes for citizens to set up such commons, government actions can easily lead to their rapid extinction. If governments aim at supporting such institutions for collective action, they have to be aware of the vulnerability of the commons as institutions. Identifying how commons develop over time as part of a larger movement can also help us to understand the drivers and potential threats associated with these developments. The study of commons so-far has been highly focussed on case-studies of particular commons as part of larger socio-ecological systems, and on how they managed to become resilient as individual organisational units, but very little attention has been given to how the governance model that commons represent as such could develop over time. This lack in commons-studies is linked to their disciplinary background, most using contemporary cases rather than historical ones. The present study provides a much longer time-perspective, and thus also offers more empirical evidence for the resilience of the commons.

The historical commons studied here originated and developed under conditions that were different from today. Although affected by top-down regulations and large scale environmental pressures beyond the control of the commoners, they locally reaped the rewards and suffered negative repercussions of their collective governance, decisions, actions and management directly. The local scale and relatively stable composition of commoners also meant that behaviours were likely influenced by relatedness, direct or indirect reciprocity, and reputation, in addition to direct personal costs and benefits. On today’s global arena, communities and social networks are typically larger, looser, and less stable. In addition, the causal links between actions—or lack thereof—and consequences are increasingly separated in space and time, and therefore more easily questioned or ignored. These are severe challenges for resource governance [13, 83].

## Methods

### Dutch commons and study area

There are two major types of Dutch shared resource self-governed commons. *Marken*, which were essentially associations of farmers, some of which have boards or steering committees, with decision making taking place at regular (yearly, or more frequent) meetings, sometimes with compulsory attendance. *Meenten*, were instead owned and managed by the entire village, and connected to the municipality. In general, however, Dutch commons were independent, each governed by an organization especially created for the purpose of the management of the collective resources and solely responsible for that. The Dutch markegenootschappen studied here were mainly (about 78 percent) located in the area of the current provinces of Overijssel, Gelderland, and Drenthe [59] (Fig 2).

The current provinces of Overijssel, Gelderland, and Drenthe, where the Dutch markegenootschappen were mainly located, are characterized by most sandy soils, in most cases needing fertilization to be cultivated, requiring mixing soil with extensive amounts of sods harvested from other uncultivated areas: for fertilizing 1 area of land, sods from 20 equivalent areas were needed. The boundaries of the area where markegenootschappen existed can partly be linked to natural boundaries: most of the eastern part of the area lies along the Veluwe ridge of hills, a formerly densely wooded area that already had been deforested by man during the Middle Ages and hence had become an area of mainly drifting sands, offering little means of existence to farmers. The northwestern boundary is formed by the so-called Hondsrug, a distinctive glacial deposit area, north of which not only floodings from the Waddensea and connecting rivers occurred very frequently, but also extensive peat bogs were found that were exploited extensively from the early 1600s onwards, making the area further north less suitable for farming. The spread pattern of the commons also indicates other natural aspects: the river bed of the IJssel river as well as the very inaccessible swamp areas in the northern part of Overijssel can be recognized from the spread pattern; inaccessibility and very low population density seem also to have determined the northwestern boundary of the area. The large rivers to the south formed also a natural border of the markegenootschappen area. Socioeconomic differences may also have played a role in setting the boundaries of the markegenootschappen area. North of this area, a large part of the land was owned either by the church, or by local noblemen; this was also the cause in the area south of the markegenootschappen area. Although most commons were located west of the Veluwe hill ridge, a small strip of commons seems to follow the most western stretches of these glacial hill ridge, ending at the common Het Gooi, which has been a very active and resilient common, existing until 1972.

### Sources of data

Information on the general large scale temporal distributions of births (*n* = 342, known year), deaths (*n* = 415) and lifespans (*n* = 351, based on either known (*n* = 342) or estimated (*n* = 581) year of birth and year of dissolution) was obtained for all commons from the Netherlands with known or estimated year of origin and dissolution within a particular area in the north-eastern part of the Netherlands from the database recently built as part of the Common Rule(s) Project (CRP) [12, 84]. The database contains over 800 commons that emerged in the same period and in the same region of the Netherlands, but information on both start and end date is only available for a subset of these, which was used for the analysis of large scale patterns. The database contains a transcription of background information taken from the original archival sources regarding the rules of use, governance and management that were designed for the commons.

In addition to exploring the larger scale patterns of births and deaths of commons, we performed more in-depth analyses of temporal dynamics in the number, spatial distribution, density, and dispersion of commons. The geographic location (coordinates) of the commons, or of the town or village the common was affiliated with, was determined to the nearest km according to Google Maps. To quantify, characterize, describe and analyse temporal trends, we calculated the number of commons, the total area occupied by commons (estimated with minimum convex polygons (MCP) which are widely used in biology to estimate the size of individuals’ home ranges [68] and species’ distribution ranges [40]). This was done at 50 year intervals, from 1050–1900. With this approach, temporal trends in MCP can inform about range size, range expansions and range contractions—not of individual commons but of the area covered by the ‘pool’ of commons. In addition, we calculated the mean distance between commons, the mean nearest neighbour distance between commons, and the variance of nearest neighbour distance between commons, also at 50 year intervals. These last measures can be used to inform whether and how the spatial dispersion of commons (e.g., uniform, random or clumped) changes over time.

### Statistical analysis

Statistical analyses were performed with SAS version 9.4. Maps were drawn using the *R* 3.4.4 platform [85] using the *ggmap* package. Kernel density estimates were performed based on the *kde2d* function in the *MASS* package.

A series of analytical steps were used to evaluate and visualize the relationship between the lifespan of commons and the number of resource types that they used. Information required to estimate both life span and the number of resource types that were managed for collective use by the commoners via rules and regulations was available for 182 of the commons. The data was analysed using multiple regression as implemented with procedure REG in SAS. Year of dissolution (death) was set as response variable, year of origination was included as a covariate (to account for the fact that lifespan is constrained by year of origination), and number of resources and squared number of resources was included in the model to test for linear and curvilinear (quadratic regression) relationships with resource use. To visualize the association of lifespan with resource use, we estimated residual lifespan for each common, as the deviations (residuals) from the least-squares linear regression linking lifespan to year of origination (*F*_1,180_ = 1157.3, *R*^2^ = 0.87, *p* < 0.0001, Fig 6a). Next, we plotted residual lifespan against the number of resource types used for each common (Fig 6b), and statistically evaluated the association using non-parametric Spearman correlation analysis. To further evaluate the evidence for a curvilinear effect of multiple resource use on residual lifespan we performed a randomization test procedure for quadratic regression. To this end, the observed values of residual lifespan were randomly reallocated to the number of resource type values, a 2-tailed test for a non-zero quadratic effect was conducted, and the outcome of 2000 randomizations was used to compute an overall significance level using the SAS macro RTEST [86].

## Supporting information

S1 Table Political, socio-economical, demographical and environmental pressures on Dutch commons. (PDF)

S2 Table Legislation regarding division of Dutch commons. (PDF)

S3 Table Data on commons used for statistical analyses. File with information provided on separate sheets for: year of origination of commons; year of dissolution of commons; lifespan of commons as a function of year of origin; year of origin and death together with geographic coordinates of commons; lifespan of commons as a function of the number and types of resources used; changes in human population size over time; and temporal changes (by 50-year intervals) in the number, density, dispersion and total area occupied by commons.
(XLSX)

S1 Movie Visual representation of the spatiotemporal dynamics of historical Dutch commons. Animation showing changes in number, spatial distribution, density and dispersion of historical Dutch commons from the 10^th^ to the 19^th^ century.
(MPG)
