# Supplementary material for: Eco-evolutionary perspectives on emergence, dispersion and dissolution of historical Dutch commons
Source: PLoS One. 2020 Jul 30;15(7):e0236471. doi: 10.1371/journal.pone.0236471 (PMC7392261; doi:10.1371/journal.pone.0236471)
Supplement: S1 Table — (PDF) [file pone.0236471.s001.pdf]

S1 Table. Overview of political, socio-economical and environmental threats to Dutch commons between the 8th and the middle of the 19th century.

8th century	Start continuous occupation city of Deventer
11th century	Foundation of cities of Kampen en Zwolle; parts of Overijssel (Oversticht) befell under episcopal rule of the bishop of Utrecht
Middle Ages	Cities claim to be independent cities (Rijkssteden), oppose episcopal rule. Rural areas mainly controlled by lesser noblemen, often conquering parts of land from other noblemen
1456	David of Burgundy became bishop of Utrecht, strove for unification of separate parts (drostambten) of Overijssel.
1456-1526	Attempts for unification by House of Burgundy lead to increased and combined opposition of cities and noblemen against episcopal rule > increased influence of 333rulers of neighboring province of Gelre.
1475-1625	Relative de-urbanization: from 38.0 to 27.9 percent of total Overijssel population. Main changes for Twente 21.2 > 25.5 and Vollenhove 9.5 > 13.9. Salland stable at 31.3 > 32.7. Slicher van Bath 1944, p. 55 [1]
1528	Rule of Overijssel transferred from Henry II, bishop of Utrecht to civil ruler Charles V. First mentioning of current name Overijssel.
1568	Start Dutch Revolt against Spanish rule. Province Overijssel supports Spanish King.
1570	Plundering rural areas by rebellious troups (Geuzen)
1576	Overijssel supports Pacification of Ghent on condition Catholic faith remains unaffected.
1578	Conquest of city of Deventer by Dutch troups. Province of Overijssel reluctantly joins Dutch Republic.
1578-1580	Soldiers of Dutch troups do not receive (sufficient) wages and start plundering rural areas.
1580	January: Overijssel farmers unite to battle against plunderers. Battle of Raalte, killing of about 700 farmers.
	June : farmers supported by Spanish troup defeat Dutch troups in Battle of Hardenbergerheide, killing about 1,600 Dutch soldiers.
1580-1597	Times of struggles and pillaging. Behavior of Dutch troups leads to large parts of rural areas remaining Catholic rather than joining Protestantism.
1585-1605	Protestantism hardly supported by population, main part of Overijssel population remains Catholic.
1597	Dutch prince Maurits finally defeats Spanish troups. Overijssel joins (Reformed) Republic.

1601-1723	Population increase; for instance, population of village of Raalte within marke Raalterwoold doubled from 235 to 470 households Slicher van Bath 1944, p. 30 [1]; proportional increase of impoverished families within Salland region (16.9 > 19.8 percent) Slicher van Bath 1944, p. 33 [1]
1601-1833	Especially change in crops: 1601 85% land, 15% meadows, in 1833: 42 and 58, Slicher van Bath 1944, p. 412 [1]
1665	First attack by troups from Münster on behalf of Holy Roman Empire. Pillaging of rural areas, mainly Twenthe.
1672	Second attack by Münster troups. Province of Overijssel is forced to denunciate alliance with Republic and to acknowledge rule of Holy Roman Empire
1674	Province of Overijssel liberated from Münster rule, readmitted to Dutch Republic under strict conditions, leading to increased power of Dutch stadhouders within Overijssel.
1675	Flood of all Saints Day (Allerheiligenvloed) – flooding of Mastenbroek polder.
1675-1795	Total population Overijssel 1675 > 79,229, 1723: 106,934, 1795: 134,104. Slicher van Bath 1944, p. 52-53 [1]
1713	Cattle plague kills about 90 percent of all cattle in the Low Countries.
1740	Very severe winter*
1750-1830	Area for agriculture multiplies 3.5. Slicher van Bath 1944, p. 407 [1]
1763	Very severe winter
1769-1770	Cattle plague, killing about 70 percent of all cattle, mainly in northern provinces.
1784	Very severe winter
1789	Very severe winter
1795	Very severe winter
1799	Very severe winter
1803	Very severe winter
1813	Very severe winter
1814	Very severe winter
1815	Rule of King William I. Increased attention to industry: metal industry and textile industry. Improvement of infrastructure, construction of transport canals.
1825	Storm surge, causing flooding in entire northwestern part of Overijssel, causing 305 fatalities.
1830	Very severe winter
1838	Very severe winter
1845	Very severe winter

1845-1846	Peek years of European Potato Failure, harvest of potatoes in Northern Netherlands decreased by 57 percent, of rye by 47 percent, of wheat by 6 percent.

Supporting reference

1. Slicher van Bath BH. Mensch en land in de Middeleeuwen. Bijdrage tot een geschiedenis der nederzettingen in oostelijk Nederland. Assen: Van Gorcum; 1944.

* Data on winters are available from 1740 and are available from website Meteolink, https://www.meteolink.nl/weerhistorie-2/historische-winters/
