# Supplementary material for: Eco-evolutionary perspectives on emergence, dispersion and dissolution of historical Dutch commons
Source: PLoS One. 2020 Jul 30;15(7):e0236471. doi: 10.1371/journal.pone.0236471 (PMC7392261; doi:10.1371/journal.pone.0236471)
Supplement: S2 Table — (PDF) [file pone.0236471.s002.pdf]

S2 Table. Legislation regarding division of Dutch commons.

**Consequences of French occupation and legislation (1795-1811)**
Commoners assemblies lose part of their public-civic rights.
**Royal Decree of 16 April 1809 (French occupation)**
Common land that has been divided and cultivated, will be exempt from land tax for the next 50 years.
**Royal Decree of 10 May 1810 (French occupation)**
Uncultivated common land should be divided unless division is impossible or harmful.
All commons are required to appoint a committee to implement the Decree.
All commons should take good care of the poor users of the commons.
**Law on tax exemption of newly cultivated land 1812**
Land tax exemption of newly cultivated land for the next 10 years.
**Cadastral implementation 1832**
Land being measured, ownership registered, and value estimated.
**Royal Decree of 28 June 1837**
Re-enforcing the Royal Decree of 1810, now easier because of cadastral registration.
**Regulation on tax exemption 1840**
Supporting Royal Decree of 1837 with tax exemption for newly cultivated land.
**Law on tax exemption of newly cultivated land 1848**
Land tax exemption of newly cultivated land for the next 20 years, after that 50% land tax exemption for the next 20 years.
**Directive Ministry of Finance 1848**
Directive resulted in lower costs for services delivered by Cadaster and land surveyors.
